# LncRNA HOTAIR regulates cell invasion and migration in endometriosis through miR-519b-3p/PRRG4 pathway

**DOI:** 10.3389/fonc.2022.953055

**Published:** 2022-10-21

**Authors:** Qiufang Bao, Qiaomei Zheng, Shaoyu Wang, Wenlu Tang, Bin Zhang

**Affiliations:** ^1^ Department of Obstetrics and Gynecology, The First Affiliated Hospital of Fujian Medical University, Fuzhou, Fujian, China; ^2^ Department of Obstetrics and Gynecology, The First Clinical College of Fujian Medical University, Fuzhou, Fujian, China

**Keywords:** endometriosis, LncRNA HOTAIR, miR-519b-3p/PRRG4, invasion, migration

## Abstract

Endometriosis is a common benign disease in gynecology and has malignant biological behaviors, such as hyperplasia, invasion, metastasis, and recurrence. However, the pathogenesis of endometriosis remains unclear. The present study aimed to investigate whether LncRNA HOTAIR regulates cell invasion and migration in endometriosis by regulating the miR-519b-3p/PRRG4 pathway. The qRT-PCR results showed that the average relative expression of LncRNA HOTAIR was much higher in ectopic endometrial tissues than in eutopic endometrial tissues. Scratch and transwell assays showed that the cell migration and invasion ability of LncRNA HOTAIR overexpression group was significantly higher than those in the control group. Conversely, the LncRNA HOTAIR knockdown group showed the opposite results. Bioinformatics analysis suggested that the downstream target genes of LncRNA HOTAIR were miR-519b-3p and *Prrg4*. Knockdown of LncRNA HOTAIR can reduce the up-regulation of *Prrg4* by miR-519b-3p and then inhibit the invasion and migration ability of endometrial stromal cells. In Conclusion, LncRNA HOTAIR can regulate the ability of invasion and migration of endometrial stromal cells, and its mechanism is proved by regulating the miR-519b-3p/PRRG4 pathway.

## Introduction

Endometriosis is a common benign disease in gynecology and has malignant biological behaviors, such as hyperplasia, invasion, metastasis, and recurrence (1–3). The main clinical features of endometriosis are dysmenorrhea, infertility, and menstrual irregularities (4). At present, the hypothesis of endometriosis includes the theory of retrograde menstruation, metaplasia of the coelom, vascular and lymphatic metastatic spread, epithelial mesenchymal transformation, altered inflammatory response, genetic susceptibility immunity, endometrial determination, and so on (4–6). However, the molecular pathogenesis of endometriosis is still not fully clarified.

Long non-coding RNAs (LncRNAs) have a length >200 nucleotides and have no protein-coding function. Recently, LncRNAs have been proved to play important roles in cell proliferation, differentiation, apoptosis, and metastasis (7). Emerging evidence has demonstrated that LncRNAs may have the potential to influence the development and persistence of cancer and endometriosis by modulating inflammation, proliferation, giogenesis, and tissue remodeling (8). LncRNA HOTAIR (Homeobox transcript antisense RNA) is highly expressed in cervical cancer, endometrial cancer, and other cancers which is proved to be related to the occurrence and metastasis of tumor (9, 10). The overexpression of LncRNA HOTAIR is associated with tumor invasion, progression, metastasis, and poor prognosis (11). Recent research showed that the expression level of LncRNA HOTAIR was elevated in endometriosis. Zhang et al. (12) found that the expression level of LncRNA HOTAIR was elevated in ectopic lesions of endometriosis. Chang et al. (13) found that patients with aggressive endometriosis expressed higher levels of LncRNA HOTAIR. Genetic alterations in LncRNA HOTAIR may be one of the risk factors leading to endometriosis development. However, the specific mechanism of LncRNA HOTAIR in the pathogenesis of endometriosis is still unclear.

A number of studies have found that LncRNAs can act as competing endogenous RNAs (ceRNAs) to bind microRNA (miRNA), regulate cell function, mediate the invasion and metastasis of cells (14). miRNAs are a class of 22 nucleotide non-coding small RNA molecules, which can bind to the 3′ untranslated regions of target gene mRNA mainly through base complementation. Moreover, they can regulate gene expression at the post-transcriptional level by degrading the target mRNA or inhibiting protein synthesis. Wang et al. (15) found that LncRNA HOTAIR can regulate the expression level of miR-326 negatively to regulate proliferation and migration in lung cancer. LncRNA HOTAIR can also serve as a sponge of miR-331-3p to regulate HER2 expression in gastric cancer (16). Zhang et al. (12) reported that exosomal LncRNA HOTAIR promotes the progression and angiogenesis of endometriosis via the miR-761/HDAC1 axis. Although there are a few reports on the regulation of endometriosis by LncRNA HOTAIR, its molecular mechanism has not been fully clarified.

In this study, we found that LncRNA HOTAIR was much higher in ectopic endometrial tissues than in eutopic endometrial tissues, which could promote the invasion and migration ability of endometrial stromal cells. Bioinformatics analysis suggested that the downstream target genes of LncRNA HOTAIR were miR-519b-3p and *Prrg4*. Further studies indicated that LncRNA HOTAIR regulates cell invasion and migration in endometriosis by regulating the miR-519b-3p/PRRG4 pathway, which may prove to be potential markers and new targets for early diagnosis and treatment of endometriosis.

## Methods and materials

### Patients and tissue collection

Paired ectopic and eutopic endometrial tissues were collected from 20 patients with ovarian endometriotic cysts, and normal endometrial tissues were collected from 10 patients without endometriosis who had surgery for other benign ovarian cysts between April 2020 and November 2020. Patients with endometriosis were pathologically confirmed. All patients between 20 and 40 years of age were examined in the Department of Gynecology and Obstetrics at the First Affiliated Hospital of Fujian Medical University in Fuzhou, People’s Republic of China. All patients in the present study had regular menstrual cycles, had not taking any combined hormonal contraception for at least six months prior to surgery, and had no other malignant, estrogen-dependent, immune, surgical, or inflammatory diseases. Patients were not classified according to menstrual cycle (menstrual cycle was divided into proliferative period, secretory period and menstrual period). We examined all cases and grouped them according to the time of menstruation: reproductive period and secretory period. There were 9 cases of reproductive period and 11 cases of secretory period in the experimental group, and 5 cases of reproductive period and secretory period in the control group. All samples for detecting LncRNA HOTAIR, miR-519b-3b and PRRG4 were from the same patient. All specimens were immediately frozen in liquid nitrogen and stored at -80°C.

This study was approved by the Ethics Committee of the First Affiliated Hospital of Fujian Medical University, Fuzhou, Fujian, China (approval number:2020 [248]). All the patients signed an informed consent form.

### Cell culture and plasmid construction

We purified primary human endometrial stromal cells as described in previous study (12). In brief, ectopic endometrial samples from patients with ovarian endometriotic cysts (proliferative period) were cut into small pieces and digested by collagenase IV and deoxyribonuclease (Sangon Biotech, Shanghai, China) for 1 h. The tissue suspension was filtered through nylon cell strainers, and stromal cells were passed through the strainer in the filtrate. Then, the suspension was centrifuged at 1000 × g for 5 min at room temperature. This action was followed by culture in red blood cell lysis buffer for 10 min to remove erythrocytes. The separated cells were cultured in DMEM/F-12(Gibco, Grand Island, USA) supplemented with 10% fetal bovine serum (Gibco, Grand Island, USA) at 37 °C under humidified air containing 5% CO_2_. The media was replaced every two days. The purified cells were used for subsequent experiments.

The plasmid of specific small interfering RNA targeting HOTAIR(si-HOTAIR) and negative control (si-NC), the plasmid of *Prrg4* overexpression (PRRG4 OE) and the control (vector) were constructed and synthesized by Anti-HeLa Biological Technology Trade, Xiamen, Fujian, China. miR-519b-3p mimics, miR-519b-3p inhibitor, and NC inhibitor were purchased from RiboBio (Guangzhou, China).

### Cell transfection

The purified cells were cultured for 48 hours, then the plasmids of HOTAIR-OE, si-HOTAIR, and miR-519b-3p inhibitor were transfected into endometrial stromal cells by using Lipofectamine™2000 reagent according to the manufacturer’s instructions. After transfection for 48 h, qRT-PCR was used to detect the expression level of miR-519b-3p. qRT-PCR and WB analyses were conducted to detect the mRNA and protein expression levels of PRRG4.

The endometrial stromal cells were divided into six groups for transfected si-NC, si-HOTAIR, si-HOTAIR+inhibitor NC, si-HOTAIR+miR-519b-3p inhibitor, si-HOTAIR+Vector, and si-HOTAIR+PRRG4 OE by using Lipofectamine™2000 reagent according to the manufacturer’s instructions, respectively. At 48 h after transfection, qRT-PCR and WB analyses were performed to detect the mRNA and protein expression levels of PRRG4.

### RNA extraction and qRT-PCR

In brief, 50 mg of tissues were obtained for ultrasonic homogenization. Total RNA was extracted using an RNA extraction kit (Novazan, Nanjing, China) following the manufacturer’s instructions. RNA concentration was measured using spectrophotometry, and purity was evaluated by the ratio of absorbance at 260 nm to 280 nm (A260/A280). qRT-PCR analysis was performed using an RT-PCR Kit (Novazan, Nanjing, China) according to the manufacturer’s instructions. Each sample was run in triplicate. The expression level of *Prrg4* was normalized using 18s as the internal control and calculated using 2^−ΔΔCt^ method. The sequences of the qRT-PCR primers were as follows: LncRNA HOTAIR, F:5′-AATAGACATAGGAGAACAC TT-3′, R:5′-AATCTTAATAGCAGGAGGAA-3′, miR-519b-3p F:5′-CGCGAAAGT GCATCCTTTTA-3′, R:5′-AGTGCAGGGTCCGAGGTAT-3′, 18s, F:5′-AGGCGC GCAAATTACCCAATCC-3′, R:5′-GCCCTCCAATTGTTCCTCGTTAAG-3′. PRRG4, F:5′-GGGAGAAGAAGTGTTTAC-3′, R:5′-CTGGCTTCCTCATAATTG-3′.

### Western blot assay

Transfected cells were harvested and rinsed using phosphate buffered saline (PBS). Total cell protein was extracted using protein lysate of RIPA, and concentration was detected with a BCA protein assay kit (Hyclone, Logan, USA). The extracted protein was collected for sodium dodecyl sulfate–polyacrylamide gel electrophoresis (SDS-PAGE) and transferred onto the polyvinylidene fluoride membrane. The membranes were blocked with skim milk for 1 h, stored overnight with PRRG4 and GAPDH primary antibodies, cleaned with TBST, and probed with HRP-conjugated secondary antibody for 2 h at room temperature. Finally, ECL chemiluminescence solution was added to analyze the gray level of protein bands and calculate the protein level of PRRG4.

### Nucleus–cytoplasm separation assay

The PARIS™ Kit (Ambion, Austin, TX) was used for nucleus–cytoplasm separation. Approximately 5×10^6^ endometrial stromal cells were placed in nucleoplasmic separation lysis fluid for resuspension and treated on ice for 10 min, which was inverted and mixed every 1 min. After centrifugation at 11,000 r/min for 10 min at 4°C, the supernatant was taken as the cytoplasm sample and precipitated into the nucleus. The content distribution of LncRNA HOTAIR in the nucleus and cytoplasm was detected by qRT-PCR. Then, the subcellular localization of LncRNA HOTAIR was determined. U6 and 18s were used as a positive control for the expression of nuclear RNA and cytoplasmic RNA, respectively.

### Dual−luciferase reporter assay

We used TargetScan to predict the binding sites of miR-519b-3p with LncRNA HOTAIR or *Prrg4*. The gene fragments of LncRNA HOTAIR or *Prrg4* were cloned and inserted into the luciferase reporter gene PmirGLO to construct wild-type plasmids of LncRNA HOTAIR or *Prrg4* (HOTAIR-Wt, PRRG4-Wt). The mutant type plasmids of LncRNA HOTAIR or *Prrg4* (HOTAIR-Mut, PRRG4-Mut) were constructed with the mutant sequences of LncRNA HOTAIR or *Prrg4* gene fragments. The endometrial stromal cells were co-transfected with miR-519b-3p mimics and HOTAIR-Mut or HOTAIR-Wt by using Lipofectamine™2000 reagent (Thermo Fisher Scientific, San Jose, USA). The cells were then co-transfected with miR-519b-3p mimics and PRRG4-Mut or PRRG4-Wt with the same method. After 48 h in an incubator at 37°C and 5% CO_2_, the co-transfected cells were harvested. Luciferase activity was detected using the dual-luciferase assay reporter kit (Promega, Madison, WI, USA).

### Scratch assay

Cells from each group were transfected in a six-well plate and cultured until they reached 90% confluence. A wound was created with a pipette tip and cultured with serum-free DMEM/F12 medium (Gibco, Grand Island, USA). According to the images captured using an inverted microscope at 0 and 48 h, the migration ability of the cells was determined by calculating the distance to the edge of the cell scratches.

### Transwell assay

For cell invasion assays, 24-well transwell plates containing 8 μm pore size inserts was paved with 50 μL of 10 mg/mL Matrigel (BD Biosciences, San Jose, USA). Cells from each group were diluted in serum-free DMEM/F12 medium, and 200 μL of the solution containing 10000 cells was inoculated in the upper chamber. About 600 μL of 20% FBS medium was added to the lower chamber. After 48 h in the incubator, the cell culture medium was sucked up with a pipette, and cells in the upper layer were gently wiped off with a cotton swab. Cells in the lower layer were washed with PBS, fixed in 4% paraformaldehyde for 20 min, and finally stained with 1% crystal violet at room temperature for 10 min. Excess dye solution was then washed off, and the cells were photographed using a light microscope. Cells in five randomly selected fields were counted. Cell migration assays were performed using similar method but without matrigel.

### Bioinformatics analysis

GSE40186 data (https://www.ncbi.nlm.nih.gov/geo/query/acc.cgi?acc=GSE40186, miRNA expression profiles in endometriotic cyst stromal cells (ECSCs)), starBase (http://starbase.sysu.edu.cn/index.php), miRNet (http://mirdb.org/index.html), and LncBase (https://www.mirnet.ca/miRNet/faces/home.xhtml) were used to predict the downstream miRNA of LncRNA HOTAIR, and the intersection of the results was used to obtain the key miRNA. After obtaining the key miRNA, GSE86534 data (https://www.ncbi.nlm.nih.gov/geo/query/acc.cgi?acc=GSE86534,Genome-wide long non-coding RNAs and mRNAs analysis of the tissues related to ovarian endometriosis), MicroT-CDS (http://diana.imis.athena-innovation.gr/DianaTools/index.php?r=microT_CDS/index), miRDB (http://mirdb.org/index.html), mirDIP (http://ophid.utoronto.ca/mirDIP/index.jsp#r), miRTarBase (https://mirtarbase.cuhk.edu.cn/~miRTarBase/miRTarBase2019/php/index.php), and starBase were used to predict the target mRNA of miRNA, and the intersection of the results was used to obtain the key mRNA.

### Statistical analysis

All statistical analyses were performed using SPSS Statistics version 21. Data are presented as means ± standard error of the mean (SEM) or median and range. Data from multiple groups were compared using one-way ANOVA, followed by Bonferroni’s *post hoc* test. Data were considered statistically significant at P<0.05.

## Results

### Expression level of LncRNA HOTAIR in endometriosis and control patients

**Figure 1:**
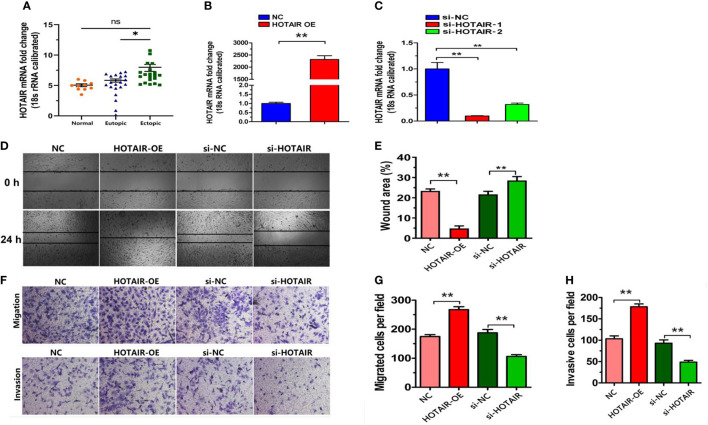
Relative expression of LncRNA HOTAIR in ectopic endometrial tissues and effect of LncRNA HOTAIR regulation on endometrial stromal cells. **(A)** Relative expression of LncRNA HOTAIR in ectopic endometrial tissues. 18s RNA was used as endogenous control. Normal, normal endometrial tissues of nonendometriosis patients; Eutopic, eutopic endometrial tissues of endometriosis patients; Ectopic, ectopic endometrial tissues of endometriosis patients. Data are presented as the mean ± SEM. *P < 0.05 vs. Normal; **(B-H)**, effect of LncRNA HOTAIR regulation on endometrial stromal cells. **(B, C)** LncRNA HOTAIR expression of different groups by RT-qPCR, **(D, E)** Cell scratch assays of the effect of overexpression LncRNA HOTAIR and si-HOTAIR on endometrial stromal cell migration, **(F-H)** Transwell assays of the effect of overexpression LncRNA HOTAIR and si-HOTAIR on endometrial stromal cell invasion and migration si-NC: negative control transfected cells; siHOTAIR: Knockdown of LncRNA HOTAIR transfected cells. NC: empty vector transfected cells; HOTAIR OE: overexpression of LncRNA HOTAIR transfected cells. Data are presented as the mean ± SEM, n=8 in each group. **P < 0.01 vs. NC or si-NC.

To investigate the expression level of HOTAIR, ectopic and eutopic endometrial tissues from 20 ovarian endometriosis patients and normal endometrial tissues from 10 control patients were performed by qRT-PCR. The results suggested that the relative expression of LncRNA HOTAIR in ectopic endometrial tissues 8.45 (4.96 -11.23) was significantly higher than that in eutopic endometrial tissues 5.76(1.18-7.95) (P < 0.05). The relative expression of LncRNA HOTAIR in ectopic endometrial tissues was slightly higher than that in normal endometrial tissues 5.08 (3.24-6.55), but the difference was not significant (P =0.067) (**Figure 1A**).

### Effects of LncRNA HOTAIR on cell invasion and migration

To understand the role of LncRNA HOTAIR in regulating the migration and invasion of endometrial stromal cells, endometrial stromal cells were transfected with siRNAs designed against LncRNA HOTAIR (si-HOTAIR-1, si-HOTAIR-2), and negative control (si-NC), over expression LncRNA HOTAIR, and empty vector (NC) (used as control). The results showed that the overexpression of LncRNA HOTAIR transfected group in endometrial stromal cells exhibited significantly higher expression compared with control cells (**Figure 1B**, P < 0.01). si-HOTAIR-1 and si-HOTAIR-2 transfected group exhibited significantly lower expression in endometrial stromal cells compared with control cells. si-HOTAIR-1 showed better effects, so si-HOTAIR-1 was used for subsequent experiments (**Figure 1C**, P < 0.01). The results indicated that the transfection was successful and used for subsequent experiments.

The cell scratch assays showed that the cell distance of endometrial cells in the overexpression LncRNA HOTAIR transfected group was significantly smaller than that in the control group (NC). The cell distance of endometrial cells in the si-HOTAIR transfected group was significantly larger than that in the control transfected group (si-NC) (**Figures 1D, E**). The transwell assays of invasion and migration showed that the number of transmembrane cells in the overexpression LncRNA HOTAIR transfected group was significantly higher than that in the control group (NC). The number of transmembrane cells in the si-HOTAIR transfected group was significantly lower than that in the control group (si-NC) (P < 0.01; **Figures 1F–H**). These results suggest that regulating LncRNA HOTAIR expression may alter the migration and invasion ability of cells.

### Downstream target genes of HOTAIR were predicted by bioinformatics analysis

**Figure 2:**
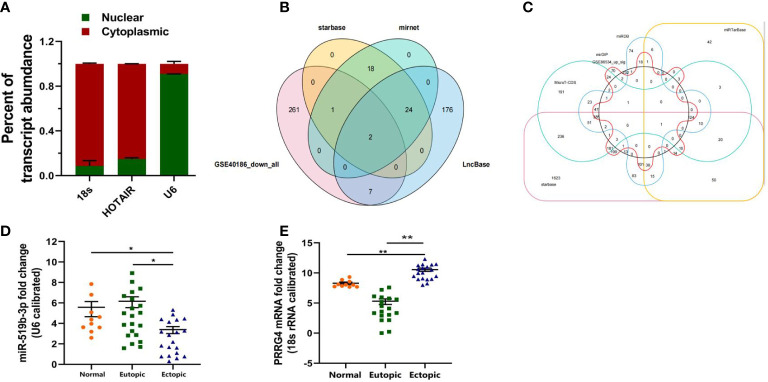
Downstream target genes of LncRNA HOTAIR were predicted by bioinformatics analysis and examined by qRT-PCR. **(A)** Nucleus–cytoplasm separation assay was used to determine the subcellular distribution of LncRNA HOTAIR in endometrial stromal cells. **(B)** Prediction of the downstream miRNAs of LncRNA HOTAIR. **(C)** Prediction of the downstream mRNAs of miR-519b-3p. **(D, E)** Relative expression level of miR-519b-3p and PRRG4 was examined in ectopic endometrial tissues compared with that in eutopic endometrial tissues. 18s RNA was used as endogenous control. Normal, normal endometrial tissues of patients without endometriosis; Eutopic, eutopic endometrial tissues of patients with endometriosis; Ectopic, ectopic endometrial tissues of patients with endometriosis. Data are presented as the mean ± SEM. *P<0.05, **P<0.01 vs. Normal.

To examine the subcellular localization of LncRNA HOTAIR and whether it could act as a miRNA sponge in endometriosis, we determined the subcellular localization of LncRNA HOTAIR using nucleus–cytoplasm separation. The results of qRT-PCR showed that the expression of LncRNA HOTAIR in the cytoplasm was higher than that in the nucleus (P < 0.05) (**Figure 2A**). The expression of LncRNA HOTAIR was mostly located in the cytoplasm. 18s was mostly located in the cytoplasm, while U6 was mostly located in the nucleus. These finding suggests that LncRNA HOTAIR mainly located in the cytoplasm which may play a regulatory role in the pathogenesis of endometriosis by binding and interacting with miRNA.

To predict the downstream miRNAs of LncRNA HOTAIR, we first screened out 271 miRNAs with low expression in endometriotic cyst stromal cells by using GSE40186 data (https://www.ncbi.nlm.nih.gov/geo/query/acc.cgi?acc=GSE40186, miRNA expression profiles in endometriotic cyst stromal cells (ECSCs)). Then, 45 miRNAs were predicted by starBase (http://starbase.sysu.edu.cn/index.php), 45 miRNAs were predicted by miRNet (http://mirdb.org/index.html), and 209 miRNAs were predicted by LncBase (https://www.mirnet.ca/miRNet/faces/home.xhtml). The four online prediction results were overlapped by Venn diagram analysis (**Figure 2B**). Two candidate miRNAs were screened out, namely, miR-519b-3p and miR-326.

We continued to predict the downstream mRNAs of miRNA. First, we predicted the downstream targeted mRNAs for miR-519B-3p. We screened out 267 mRNAs with high expression in ovarian endometriosis by using GSE86534 data (https://www.ncbi.nlm.nih.gov/geo/query/acc.cgi?acc=GSE86534,Genome-wide long non-coding RNAs and mRNAs analysis of the tissues related to ovarian endometriosis). Then, a total of 1333 mRNAs were predicted by MicroT-CDS (http://diana.imis.athena-innovation.gr/DianaTools/index.php?r=microT_CDS/index), 988 mRNAs were predicted by miRDB (http://mirdb.org/index.html), 1235 mRNAs were predicted by mirDIP (http://ophid.utoronto.ca/mirDIP/index.jsp#r), 344 mRNAs were predicted by miRTarBase (https://mirtarbase.cuhk.edu.cn/~miRTarBase/miRTarBase2019/php/index.php), and 3174 mRNAs were predicted by starBase (http://starbase.sysu.edu.cn/index.php). These online prediction results were overlapped by Venn diagram analysis (**Figure 2C**). Finally, the downstream target mRNA (PRRG4) of miR-519b-3p was identified. However, the downstream target mRNA of miR-326 was not found.

### The expression level of miR-519b-3p and PRRG4 was detected by qRT-PCR

To determine whether LncRNA HOTAIR can regulate endometrial stromal cells by sponging miR-519b-3p, we detected the expression level of miR-519b-3p in patients with endometriosis and control patients by qRT-PCR. The results suggested that the relative expression of miR-519b-3p in ectopic endometrial tissues 3.75(0.43-5.64) was significantly lower than that in eutopic endometrial tissues 6.06 (1.58-9.34) (P < 0.05). The relative expression of miR-519b-3p in ectopic endometrial tissues was lower than that in normal endometrial tissues 5.72(2.36-7.95). The differences were all statistically significant (P < 0.05) (**Figure 2D**).

To investigate the expression level of PRRG4, we evaluated the ectopic and eutopic endometrial tissues from 20 patients with ovarian endometriosis and 10 control patients with normal endometrial tissues by qRT-PCR. The relative expression level of PRRG4 in ectopic endometrial tissues 11.06(7.13-13.16) was significantly higher than that in eutopic endometrial tissues 5.36(0.02-7.89). The relative expression level of PRRG4 in ectopic endometrial tissues was significantly higher than that in normal endometrial tissues 8.25(7.12-9.67). The differences were all statistically significant (P<0.01, **Figure 2E**).

### Downregulating the level of LncRNA HOTAIR increased the expression level of miR-519b-3p

**Figure 3:**
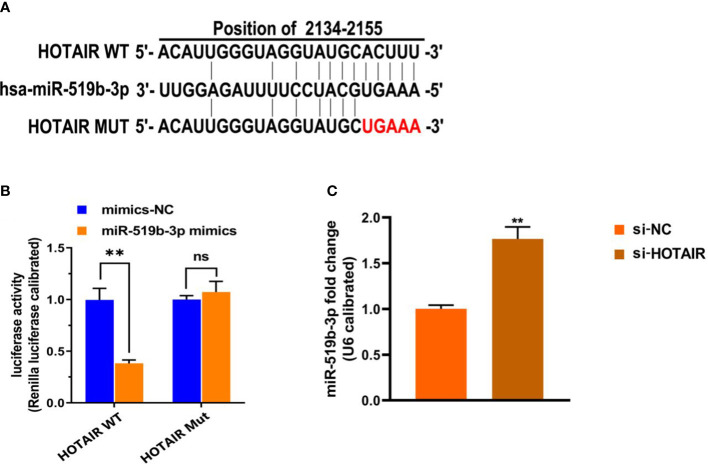
miR-519b-3p was a target gene of LncRNA HOTAIR and negatively regulated by LncRNA HOTAIR. **(A)** Predicted complementary sequences between LncRNA HOTAIR and miR-519b-3p, **(B)** Luciferase reporter assay confirmed that the luciferase activity decreased when LncRNA HOTAIR was bound to miR-519b-3p-wt, **(C)** Expression level of LncRNA HOTAIR was detected by qRT-PCR. Data are presented as the mean ± SEM, n=8 in each group. **P<0.01 vs. mimics-NC or si-NC. ns, not significant.

To further explore the underlying mechanism of LncRNA HOTAIR in endometriosis, we used TargetScan to predict whether miR-519b-3p might be the target gene of LncRNA HOTAIR (**Figure 3A**). Dual-luciferase reporter assay was used to validate the targeting relationship between LncRNA HOTAIR and miR-519b-3p. The transfection with miR-519b-3p mimics significantly repressed the luciferase activity of HOTAIR-wt in endometrial stromal cells (P<0.01) but had no effect on the luciferase activity of HOTAIR-mut (**Figure 3B**). Thus, HOTAIR may mediate the degradation of miR-519b-3p in endometriosis. Subsequently, to determine whether miR-519b-3p expression is affected by LncRNA HOTAIR, we transfected endometrial stromal cells with si-NC and si-HOTAIR and detected miR-519b-3p expression by qRT-PCR. The knocked down of LncRNA HOTAIR significantly increased the expression level of miR-519b-3p (P<0.01, **Figure 3C**). These results suggested that miR-519b-3p was the target gene of LncRNA HOTAIR and negatively regulated by LncRNA HOTAIR.

### Inhibiting the level of miR-519b-3p increased the expression level of PRRG4

**Figure 4:**
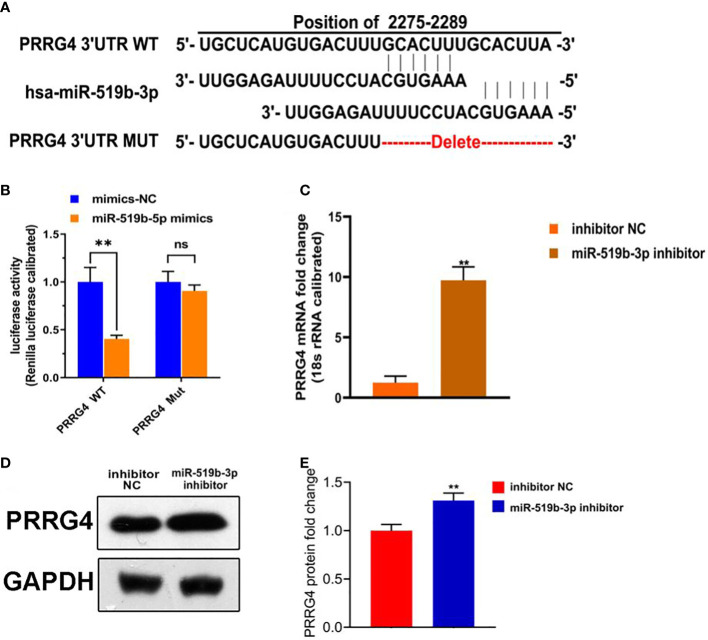
miR-519b-3p was a target gene of LncRNA HOTAIR and negatively regulated by LncRNA HOTAIR. **(A)** Predicted complementary sequences between miR-519b-3p and PRRG4, **(B)** Luciferase reporter assay confirmed that the luciferase activity decreased when miR-519b-3p was bound to PRRG4-wt, **(C-E)** mRNA and protein expression levels of PRRG4 were detected by qRT-PCR and WB. Data are presented as the mean ± SEM, n=8 in each group. **P<0.01 vs. mimics-NC or inhibitor NC.

To further explore the underlying mechanism of miR-519b-3p in endometriosis, we used TargetScan to predict whether *Prrg4* might be the target gene of miR-519b-3p (**Figure 4A**). Dual-luciferase reporter assay was used to detect the targeting relationship between miR-519b-3p and PRRG4. The transfection with miR-519b-3p mimic significantly repressed the luciferase activity of PRRG4-wt in endometrial stromal cells (P<0.01) but had no effect on the luciferase activity of PRRG4-mut (**Figure 4B**). Thus, miR-519b-3p may mediate the elevation of PRRG4 in endometriosis. Subsequently, to determine whether PRRG4 expression is affected by miR-519b-3p, we transfected endometrial stromal cells with inhibitor NC and miR-519b-3p inhibitor and detected PRRG4 expression by qRT-PCR and WB. The result showed that miR-519b-3p significantly increased the mRNA and protein expression levels of PRRG4 (P<0.01, **Figures 4C–E**). Collectively, these results suggested that *Prrg4* was a target gene of miR-519b-3p and negatively regulated by miR-519b-3p.

### LncRNA HOTAIR affected the biological behavior of endometrial stromal cells by regulating miR-519b-3p and PRRG4

**Figure 5:**
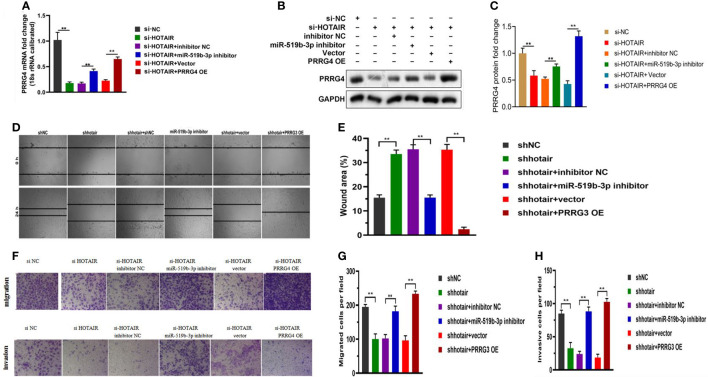
LncRNA HOTAIR combined with miR-519b-3p to regulate PRRG4 expression and migration and invasion ability of endometrial stromal cells. **(A-C)** Endometrial stromal cells were transfected with si-NC, si-HOTAIR, si-HOTAIR+inhibitor NC, si-HOTAIR+miR-519b-3p inhibitor, si-HOTAIR+Vector, and si-HOTAIR+PRRG4 OE. mRNA and protein levels of RPRG4 were examined by qRT-PCR and WB. **(D-H)** Scratch and transwell assays were used to detect the migration and invasion ability of endometrial stromal cells in different groups. Data are presented as the mean ± SEM, n=8 in each group. **P<0.01 vs. si-NC or shNC.

To clarify the relationship among LncRNA HOTAIR, miR-519b-3p, and PRRG4 in endometriosis, we conducted a rescue experiment. The transfected endometrial stromal cells were divided into the following groups: cells in the si-NC group were transfected with the negative control plasmid (si-NC), cells in the si-HOTAIR group were transfected with si-HOTAIR plasmid, cells in the si-HOTAIR+inhibitor NC group were co-transfected with si-HOTAIR plasmid and inhibitor NC, cells in the si-HOTAIR+miR-519b-3p inhibitor group were co-transfected with si-HOTAIR plasmid and miR-519b-3p inhibitor, cells in the si-HOTAIR+Vector group were co-transfected with si-HOTAIR plasmid and empty plasmid for PRRG4, and cells in the si-HOTAIR+PRRG4 OE group were co-transfected with si-HOTAIR plasmid and overexpression PRRG4 plasmid. The mRNA and protein levels of PRRG4 were all reduced by si-HOTAIR in endometrial stromal cells, while down-regulating miR-519b-3p or up-regulating PRRG4 reversed the downregulation of PRRG4 levels (P<0.01, **Figures 5A–C**). These finding suggested that LncRNA HOTAIR can competitively bind to miR-519b-3p to regulate the expression level of the target gene PRRG4, consistent with the ceRNA regulatory network predicted by bioinformatics analysis. The results of scratch assays (**Figures 5D, E**) and transwell assays (**Figures 5F–H**) showed that si-HOTAIR decreased the invasion and migration ability of endometrial stromal cells, whereas these effects were partially reversed by the down-regulation of miR-519b-3p or the up-regulation of PRRG4. Hence, miR-519b-3p inhibitor or overexpression of PRRG4 could partially reverse the downregulation effects of si-HOTAIR on the migration and invasion of endometrial stromal cells. Based on these results, LncRNA HOTAIR could combine with miR-519b-3p to regulate the expression level of PRRG4 and then regulate the migration and invasion behaviors of endometrial stromal cells.

## Discussion

Endometriosis is defined as presence of growing–functioning endometrial tissue outside the uterine cavity, and its specific pathogenesis is still unclear. Many hypotheses have been established for endometriosis, but none of them can fully explain its etiology and developmental mechanism (4, 5). Menstrual reflux/planting is the generally accepted hypothesis that the endometrial tissue of the menstrual period flows to the abdominal cavity through the fallopian tube, and then migrates and implants to form ectopic lesions. During this process, the reflux endometrium can form ectopic lesions through a series of processes such as adhesion, invasion, and migration and then develops into endometriosis. This disease has biological behaviors that similar to cancer such as proliferation, invasion, and metastasis of recurrent malignant tumors.

Long non-coding RNAs (lncRNAs) are a class of RNAs longer than 200 nucleotides with no protein-coding function. Studies have shown that LncRNA plays an important role in the proliferation, differentiation, apoptosis, and metastasis of cancer cells, leading to tumor invasion, progression, metastasis, and poor prognosis. Scholars have focused on the role of LncRNA in endometriosis. Sun et al. (17) first performed microarray analysis of LncRNA expression in ovarian endometriosis and found that 948 LncRNA transcripts and 4088 mRNA transcripts were dysregulated in ectopic endometrial tissue compared with those in paired eutopic endometrial tissue. Wang et al. (14) discovered that LncRNAs are differentially expressed between ectopic and eutopic endometrial tissues, and some dysregulated LncRNAs can be used as non-invasive biomarkers for diagnosis of endometriosis. Ghazal et al. (18) proved that the expression level of H19 was significantly decreased in patients with endometriosis and may be involved in regulating the pathogenesis of infertility. HOX transcript antisense intergenic RNA (HOTAIR) is one of the most studied LncRNAs. It is transcribed from the antisense strand of the HOXC gene cluster and influences the expression of genes from the HOXD locus. LncRNA HOTAIR, with length of about 2200 nt, was located on chromosome 12q13.13 between HOXC11 and HOXC12 genes. Dysregulations of LncRNA HOTAIR were often involved in the occurrence and development of malignant tumors and were associated with tumor invasion, progression, and metastasis. LncRNA HOTAIR overexpression was found in patients with breast cancer, and its overexpression was associated with metastasis and poor prognosis (19). Qiu et al. (20) showed that LncRNA HOTAIR was highly expressed in ovarian cancer, which was positively correlated with the invasion ability of cells, and its overexpression was associated with invasive metastasis and poor prognosis. Zhang et al. (12) showed that the ectopic endometrium exhibited higher expression of HOTAIR compared with that in paired eutopic endometrium and normal endometrium. In this study, we found that the LncRNA HOTAIR expression increased in endometriosis, and the LncRNA HOTAIR level of ectopic endometrial tissues was significantly higher than that of eutopic endometrial tissues, thereby promoting the ability of migration and invasion in endometrial stromal cells and playing an important role in the pathogenesis of endometriosis.

MicroRNAs (miRNAs), another class of endogenous non-coding RNAs with approximately 22 nucleotides in length, can bind to the 3′ untranslated regions (3′ UTR) of target genes to modulate gene expression mainly through base complementation. LncRNAs can act as competing endogenous RNAs (ceRNAs) to combine with miRNAs to regulate the downstream target mRNA and then play biological functions (21). Wang et al. (22) found that LINC00261 can act as ceRNA to regulate BCL2L11 expression by combining with miR-132-3p and participating in the invasion of endometriosis. Liu et al. (23)found that LncRNA-H19 could inhibit ectopic endometrial cell proliferation and invasion by modulating miR-124-3p and ITGB3. Many studies suggested that LncRNA HOTAIR can also be used as ceRNA to combine with target miRNAs and regulate cell functions. LncRNA HOTAIR can regulate cell migration and invasion through miR-152-3p6 in endometrial carcinoma (24). It can attenuate the inhibitory effect of miR-129-5p on cervical cancer cells and may be involved in the regulation of the progression of cervical cancer (25). Zhang et al. (12) reported that exosomal LncRNA HOTAIR promotes the progression and angiogenesis of endometriosis via the miR-761/HDAC1 axis. In the present study, we found that LncRNA HOTAIR could competitively bind to miR-519b-3p to control the expression level of miR-519b-3p. Decreasing the LncRNA HOTAIR expression can increase the expression level of miR-519b-3p and then regulate the invasion and migration ability of endometrial stromal cells. This result also suggests that LncRNA HOTAIR may act on multiple miRNAs to participate in the occurrence of diseases.

Proline-rich γ-carboxyglutamic acid protein 4 (PRRG4) is one of the cell surface transmembrane proteins in the PRRG family that has γ-carboxyglutamate (Gla) residues at the extracellular site and WW binding motifs in the cytosole. Yamamoto et al. (26) showed that the gene deletion of *Prrg4* may be related to WAGR syndrome, which is an autosomal hereditary disease. A recent study suggested that PRRG4 plays an important role in the metastasis and invasion of breast cancer cells (27). Zhang et al. (12) reported that Exosomal HOTAIR promoted the progression and angiogenesis of endometriosis by regulating the miR-761/HDAC1 axis and activating STAT3-mediated inflammation *in vitro* and *in vivo*. In the present work, we found that *Prrg4* was the target gene of miR-519b-3p by bioinformatics analysis. The qRT-PCR analysis results showed that the expression level of PRRG4 increased in endometriosis, especially in ectopic endometrial tissues. Inhibiting the expression of miR-519b-3p can target up-regulate the expression level of PRRG4. Through the recovery experiment, we found that the low expression of LncRNA HOTAIR could down-regulate the expression level of PRRG4 and reduce the migration and invasion ability of endometrial stromal cells. Adding miR-519b-3p inhibitor or overexpressing PRRG4 could partially reverse the down-regulation of PRRG4 and the downregulation effects of si-HOTAIR on the migration and invasion of endometrial stromal cells. This result also suggests that LncRNA HOTAIR may affect multiple signal pathways to participate the molecular pathogenesis of endometriosis.

In conclusion, LncRNA HOTAIR elevated the expression level of PRRG4 by sponging miR-519b-3p to promote the invasion and migration ability of endometrial stromal cells. The LncRNA HOTAIR/miR-519b-3p/PRRG4 pathway was involved in the pathogenesis of endometriosis and might be a potential marker and new target for early diagnosis and treatment of endometriosis.

## Data availability statement

The original contributions presented in the study are included in the article/supplementary material. Further inquiries can be directed to the corresponding author.

## Ethics statement

The studies involving human participants were reviewed and approved by Ethics Committee of the First Affiliated Hospital of Fujian Medical University. The patients/participants provided their written informed consent to participate in this study.

## Author contributions

QB carried out most of the experiments and analyzed the data. BZ conceived and supervised the project, provided suggestion to the experiments, discussed the data and wrote the manuscript with contributions from QZ, WT, and SW. All authors contributed to editing the manuscript.

## Funding

This work was supported by Scientific Research Project from the Education Department of Fujian Province (No. JAT190208) and Natural Science Fundation of Fujian Province (No.2022J01218).

## Conflict of interest

The authors declare that the research was conducted in the absence of any commercial or financial relationships that could be construed as a potential conflict of interest.

## Publisher’s note

All claims expressed in this article are solely those of the authors and do not necessarily represent those of their affiliated organizations, or those of the publisher, the editors and the reviewers. Any product that may be evaluated in this article, or claim that may be made by its manufacturer, is not guaranteed or endorsed by the publisher.
